# Assessment of Lumbar Vertebrae L1–L7 and Proximal Femur Microstructure in Sheep as a Large Animal Model for Osteoporosis Research

**DOI:** 10.3390/biology14081031

**Published:** 2025-08-11

**Authors:** José A. Camassa, Vera V. Barros, Pedro S. Babo, Fábio A. M. Pereira, José J. L. Morais, Aureliano Fertuzinhos, Jorge T. Azevedo, Rui L. Reis, Manuela E. Gomes, Ana Martins-Bessa, Carlos A. Viegas, Sílvio H. de Freitas, Nuno Dourado, Isabel R. Dias

**Affiliations:** 1 Department of Veterinary Sciences, School of Agricultural and Veterinary Sciences (ECAV), University of Trás-os-Montes e Alto Douro (UTAD), Quinta de Prados, 5000-801 Vila Real, Portugal; camassa.vet@gmail.com (J.A.C.); abessa@utad.pt (A.M.-B.); cviegas@utad.pt (C.A.V.); 2 Headquarters of the European Institute of Excellence on Tissue Engineering and Regenerative Medicine, 3B’s Research Group, I3Bs—Research Institute on Biomaterials, Biodegradables and Biomimetics, University of Minho, AvePark, Rua Ave 1, Edificio 1 (Sede), Barco, 4805-694 Guimarães, Portugal; verinha_b.94@hotmail.com (V.V.B.); pedro.babo@i3bs.uminho.pt (P.S.B.); rgreis@i3bs.uminho.pt (R.L.R.); mmegomes@icbas.up.pt (M.E.G.); 3 ICVS/3B’s—PT Government Associate Laboratory, Braga, 4805-017 Guimarães, Portugal; 4 Department of Engineering, School of Sciences and Technology, University of Trás-os-Montes e Alto Douro (UTAD), Quinta de Prados, 5000-801 Vila Real, Portugal; famp@utad.pt (F.A.M.P.); jmorais@utad.pt (J.J.L.M.); 5 Centre for the Research and Technology of Agroenvironmental and Biological Sciences (CITAB), Inov4Agro, University of Trás-os-Montes e Alto Douro (UTAD), Quinta de Prados, 5000-801 Vila Real, Portugal; 6 Center for Micro Electro Mechanics Systems (CMEMS), Department of Mechanical Engineering, University of Minho, Campus of Azurém, 4800-058 Guimarães, Portugal; afertuzinhos@dem.uminho.pt (A.F.); nunodourado@dem.uminho.pt (N.D.); 7 Department of Animal Sciences, School of Agricultural and Veterinary Sciences (ECAV), University of Trás-os-Montes e Alto Douro (UTAD), Quinta de Prados, 5000-801 Vila Real, Portugal; jorgemtazevedo@gmail.com; 8 Animal and Veterinary Research Center (CECAV), AL4AnimalS, University of Trás-os-Montes e Alto Douro (UTAD), Quinta de Prados, 5000-801 Vila Real, Portugal; 9 Unit for Multidisciplinary Research in Biomedicine (UMIB), School of Medicine and Biomedical Sciences (ICBAS), University of Porto, Rua Jorge Viterbo Ferreira 228, 4050-313 Porto, Portugal; 10 Vasco da Gama Research Center (CIVG), University School Vasco da Gama (EUVG), Campus Universitário, Av. José R. Sousa Fernandes, Lordemão, 3020-210 Coimbra, Portugal; 11 Departamento de Medicina Veterinária, Faculdade de Zootecnia e Engenharia de Alimentos (FZEA), Universidade de São Paulo (USP), Pirassununga 13635-900, Brazil; silviohfreitas@gmail.com; 12 LABBELS—Laboratório Associado, 4710-057 Braga, Portugal

**Keywords:** lumbar vertebrae, proximal femur, osteoporosis, sheep model, micro-computed tomography analysis, bone histomorphometry

## Abstract

Sheep have been widely used as a model for osteoporosis research. This study aimed to characterise changes in microstructure and composition in lumbar vertebrae L1–L7 and the proximal femur after implementation of a bone loss induction protocol (in this species). A sham control and experimental group (glucocorticoid-treated ovariectomized sheep) were used (*n* = 6/group), with a study duration up to the 24th postoperative week. Through micro-computed tomography, vertebrae and femoral head trabecular bones from the experimental group presented a consistent decrease in bone volume fraction (BV/TV), trabecular thickness (Tb.Th), and trabecular number (Tb.N) and an increase in trabecular separation (Tb.Sp) and total porosity (*p* > 0.05). The mineral density of the femoral heads from the experimental group showed a statistically significant decrease (*p* ˂ 0.05). The entire histomorphometric analysis of the vertebrae in the experimental group showed an increase in cortical porosity (Ct.Po) and a decrease in cortical thickness (Ct.Th) (*p* ˂ 0.0001 and *p* ˂ 0.001, respectively). Vertebrae L6 and L7 were the most affected, showing a significant increase in Ct.Po (*p* < 0.05) and a significant decrease in Ct.Th at the L6 level (*p* < 0.05). Regarding the trabecular bone at the vertebral level, only L4 showed a significant increase in Tb.Sp (*p* ˂ 0.05). In the femoral heads’ subchondral cortical layer, the Ct.Po increased significantly and Ct.Th decreased (*p* < 0.01), and at the trabecular level, the BV/TV, Tb.Th, and Tb.N decreased significantly, while Tb.Sp increased (*p* < 0.05). In conclusion, the L4, L6 and L7 vertebrae seem the most suitable for further preclinical and translational studies of vertebral augmentation or spinal fusion in this animal model.

## Simple Summary

Large animal models of osteoporosis are important for preclinical testing of new anti-osteoporotic drugs, research in biomaterials, and development of orthopaedic implants and prostheses for fixation of fragility fractures and joint replacements in the osteopenic/osteoporotic human skeleton. This study aims to evaluate alterations in micro-architectural properties at the level of the various vertebrae of the lumbar spine and the femoral head in an ovine model of osteoporosis, induced through ovariectomy and subsequent establishment of a corticosteroid administration protocol. This objective is important, because better animal models for studying osteoporosis increase the quality of the knowledge that can be obtained through their use. Most studies conducted to date using ovine models of osteoporosis focus on only one lumbar vertebra. In this study, we compare the effects of osteoporosis induction on all seven lumbar vertebrae, as well as the femoral heads, since these are two skeleton areas that typically experience the greatest bone density loss in human osteoporosis. The L4 vertebra was the most influenced by the induction of osteoporosis at the trabecular bone level, while the L6 and L7 vertebrae were the most influenced at the cortical bone level, showing these vertebrae to be the most suitable for subsequent osteoporosis studies.

## 1. Introduction

Osteoporosis is a skeletal disorder characterised by a loss of bone mass and structure and in which the bone strength is compromised, thus increasing the risk of fragility fractures [1]. This disorder can be classified as type I and type II osteoporosis. Type I, or postmenopausal osteoporosis, is mainly characterised by a loss of trabecular bone, increasing the number of fractures of the vertebrae, and typically affects women after menopause due to a lack of endogenous oestrogens [2,3]. On the other hand, type II, or senile osteoporosis, causes a loss of cortical and trabecular bone in both men and women, as it is the end result of age-related bone loss and is characterised by hip, proximal humerus, proximal tibia, and pelvis fragility fractures [2]. In a healthy skeleton, constant bone remodelling occurs, in which mature bone tissue is removed in a process called resorption, and new tissue is formed in order to maintain bone strength and mineral homeostasis in continuum, with strict coordination between their phases [4]. The bone remodelling cycle has three phases, the initiation, transition (reversal), and termination of bone formation, which have been thoroughly described in the literature [4,5,6]. The process of bone remodelling involves the osteoprotegerin (OPG)/receptor activator of nuclear factor NF-κB ligand (RANKL)/its membrane-bound receptor (RANK) system on osteoblasts and osteoclasts [7,8,9,10,11]. RANKL can be produced by numerous hematopoietic (e.g., T- and B-cells) and mesenchymal (osteoblast lineage, chondrocyte) cell types, representing an essential cytokine for bone resorption by osteoclasts [12]. The OPG and RANKL constitute a ligand–receptor system that directly regulates osteoclast differentiation, and OPG acts as an inhibitor of osteoclastogenesis by competing with RANKL for the membrane receptor [7,8,9,10,11].

Osteoporosis is caused by an imbalance in the bone remodelling process as a consequence of the miscoordination of several of the communication pathways between osteoblast and osteoclast lineages [13]. Reduced oestrogen levels induce an unbalanced remodelling, increasing bone loss and the risk of osteoporosis [14]. Osteoblast and osteoclast activities are controlled by a variety of cytokines and hormones, namely, oestrogens. Oestrogen binds with oestrogen receptors, promoting the expression of OPG and suppressing the action of RANKL [12,15]. Oestrogen can also activate Wnt/β-catenin signalling, enhancing osteogenesis, and upregulate BMP signalling, thus promoting mesenchymal stem cell (MSC) differentiation from pre-osteoblasts to osteoblasts, rather than adipocytes [15]. A decrease in oestrogen will also affect the expression of oestrogen target genes, increasing the secretion of IL-1, IL-6, and tumour necrosis factor (TNF) [15].

In terms of glucocorticoids (GCs), their excess alters the bone metabolism and decreases the bone mineral density (BMD) and strength of cortical and trabecular bone tissues, thus increasing the prevalence of atraumatic fractures, osteonecrosis, and muscle weakness [16,17]. Consistent administration of supraphysiological levels of GCs affects the canonical bone morphogenetic protein (BMP) pathway and inhibits Wnt protein production, promoting mesenchymal progenitor cells to differentiate toward adipocytes rather than osteoblasts [15]. GCs can also elevate the RANKL/OPG ratio, causing bone resorption through maturation and activation of osteoclasts, while GC excess is also associated with osteoblast and osteocyte apoptosis [15]. Within the first 3–6 months after the start of administering high daily and high cumulative GC doses, the highest rate of bone loss occurs, increasing in particular the risk of vertebral fracture due to the greater effects of GCs on trabecular bone than on cortical bone [18,19].

Despite sheep presenting relevant differences to humans in their bone tissue, especially in their macro- and microstructure, composition, biochemical properties, and bone mineral density (BMD) [20,21,22], as well as in bone metabolism (although influenced by seasonality) [23], this small ruminant has been described as an efficient animal model for osteoporosis research [21,24,25,26]. Sheep is also the recommended species to use for preclinical large animal models by the Food and Drug Administration when studying postmenopausal osteoporosis [27]. Andreasen et al. [28] concluded that GC-treated and ovariectomized (OVX) aged sheep presented with significant bone loss, promoted by an arrest of the reversal phase, resulting in an uncoupling of bone formation and resorption, as has also been demonstrated in postmenopausal women with GC-induced osteoporosis [29,30]. Another study elucidated the osteocyte regulation of OPG/RANKL in a sheep model of osteoporosis, concluding that in the late progressive phase of steroid-induced osteoporosis, the expression of RANKL is stimulated in osteocytes [31]. Therefore, the by far most frequently used small ruminant model for osteoporosis research is the OVX sheep model with 12 or more months postoperatively, or the combined GC-treated and OVX sheep model, in which the sheep are also ideally fed with a calcium-/vitamin-D-/phosphorus-deficient diet [32,33,34]. This animal model is used for preclinical testing of new anti-osteoporotic pharmacological strategies, research in biomaterials, and the development of orthopaedic implants and prostheses for fixation of fragility fractures and joint replacements in the osteopenic/osteoporotic skeleton [32].

Although several studies on the GC-treated and OVX sheep model have been published [35], none of them evaluate each of the lumbar vertebrae individually, which may contribute to verifying whether significant differences in micro-architectural and bone tissue composition occur between different vertebrae. This comparative study may help to verify which of these vertebrae are more suitable for further preclinical and translational studies of vertebral augmentation or spinal fusion in this animal model. In addition, the comparison of the bone tissue’s response, at the level of the axial and appendicular skeleton, to OVX and subsequent exogenous GC administration in this animal model is relevant for this purpose. Therefore, the present study aimed to contribute to the knowledge of the GC-treated and OVX sheep model by evaluating the micro-architectural characteristics and bone composition of all the lumbar vertebrae L1–L7 and proximal femur individually, with samples being acquired and evaluated by means of micro-computed tomography (µCT), histology, and bone histomorphometry.

## 2. Materials and Methods

This study was carried out in the Veterinary Teaching Hospital of the University of Trás-os-Montes e Alto Douro, Vila Real, Portugal (latitude 41°19′ N, longitude 7°44′ W, and altitude 479 m). All animal handling practices followed European legislation on animal experimentation and the ARRIVE guidelines.

### 2.1. Animals and Housing

Twelve healthy female Serra da Estrela sheep (*Ovis aries*) aged 8 to 9 years (mean weight of 55.95 ± 4.5 kg) were housed indoors in spacious, dry, well-drained, and ventilated boxes, with the bedding being composed of hay and straw and regularly changed. The animals were kept under a constant photoperiod cycle (light: from 07:00 to 19:00 h; dark: from 07:00 to 19:00 h), temperature (20 ± 2 °C), and humidity (50 ± 10%) in spacious cages, in groups of 6 animals. They were fed with grass hay and food pellets (0.250 kg/animal/day), and water was provided ad libitum. The diet provided an estimated 1.20× energy maintenance requirements according to the NCR [36] recommendations for sheep nutrition. The animals were acclimatised for 4 weeks before being subjected to the anaesthetic and surgical protocols.

### 2.2. Anaesthetic and Surgical Protocols

The animals were randomly assigned to a sham control and experimental group (*n* = 6/group), where the ewes were bilaterally OVX and posteriorly received 1 mg/kg dexamethasone injections weekly, as described by Lill et al. [37] and Schorlemmer et al. [38]. For OVX, the anaesthetic protocol was composed of premedication with acepromazine maleate (0.1 mg/kg IV, Calmivet; Univete, Lisbon, Portugal). The anaesthetic induction was carried out with butorphanol tartrate (0.06 mg/kg IV, Torbugesic; Fort Dodge Veterinaria, S.A., Vall de Vianya, Girona, Spain) and propofol 2% (3 mg/kg IV, Propofol-Lipuro; B.Braun, Melsungen, Germany) and maintained with 1.5% isoflurane in oxygen. Analgesia was accomplished by means of the administration of flunixin meglumine (1 mg/kg, IM, q24h, Finadyne; Vetlima, Lisbon, Portugal) for 72 h, and the animals were given anti-biotherapy with amoxicillin (15 mg/kg, IM, q48h, Clamoxyl LA; Laboratórios Pfizer, Lda, Barreiro, Portugal) during the first week. The experimental group received 1 mg/kg dexamethasone in weekly injections (0.6 mg/kg IM, Dexafort, MSD Animal Health, Portugal; 0.4 mg/kg IM, Oradexon, N.V. Organon, Oss, The Netherlands). During the last four weeks, the tapering of steroids was performed (¾, ½, ¼, and 0 of the initial steroid dose), since the complete removal of GCs it would be necessary for the subsequent use of this animal model in the study of anti-osteoporotic drugs, submission to anaesthetic protocols, or development of surgical techniques for orthopaedic implants and biomaterial research on the osteopenic/osteoporotic skeleton. All animals completed the study period with no complications and were euthanized at the 24th postoperative week with a lethal IV injection of pentobarbital sodium (100 mg/kg IV, Eutasil; Sanofi Veterinária, Miraflores, Algés, Portugal) after sedation with xylazine (0.1 mg/kg, IV, Rompun; Bayer Portugal, Lda, Carnaxide, Portugal).

### 2.3. X-Ray Micro-Computed Tomography (µCT)

Individual vertebral bodies were isolated by removing posterior elements at the pedicle, and the samples from lumbar vertebrae L1 to L7 were obtained by means of posterolateral extrapedicular lumbar vertebral biopsy and mid-sagittal biopsy to determine cortical and trabecular bone structural parameters and the cortical thickness. The samples from the femoral heads were obtained immediately above the fovea capitis femoris, as a reference point, and included cartilage, subchondral bone, and trabecular bone. The bone samples were obtained using bone dental trephines with 6 mm and 10 mm inner diameters for vertebrae and femur samples, respectively. All methods used to assess the microstructure and composition of bone samples and analysis were performed blindly. Samples from the lumbar vertebrae L1 to L7 (6 mm diameter biopsies) and from the femoral heads (10 mm diameter biopsies) were scanned using an X-ray scanning micrograph (µ-CT; SkyScan 1272; Bruecker, Kontich, Belgium). The samples were maintained under wet conditions by wrapping them with filter paper soaked in saline. Series of two-dimensional projections, with a resolution of 7 μm, were acquired over a rotation range of 180° and with a rotation step of 0.45° by means of cone-beam acquisition and using a 0.35 mm copper + 0.15 mm aluminium filter.

The data were reconstructed using the NRecon software (version 1.6.6.0, Skyscan) and analysed in a CT analyser (version 1.17.0.0, Skyscan). The region of interest (ROI) was defined as a 4.5 mm diameter circle, centred over the specimen. Based on auto-interpolation of a manually defined ROI with the inner and outer limits of the trabecular bone, we yielded a volume of interest (VOI) in the shape of a cylinder that was representative of the trabecular region of the sample, which was the main basis for the quantitative analyses. The BMD (g/cm^3^) of each sample was determined using 8 mm phantom calibrators of 0.25 and 0.75 g/cm^3^. The bone volume fraction (BV/TV; %), specific bone surface (BS/BV; %), trabecular thickness (Tb.Th; μm), trabecular number (Tb.N; 1/mm), trabecular spacing (Tb.Sp; mm), closed porosity (Po(cl); %), open porosity (Po(op); %), and total porosity (Po(tot); %) were calculated using the Batman tool of the CT analyser software. For the three-dimensional (3D) analysis, the bone region of each section was automatically defined (using the Ridler–Calvard method), and the resulting binarized image was despeckled to remove the background (for bright speckles < 40 voxels). The 3D reconstructions were produced using the CTVOX software (Version 2.5).

### 2.4. Histology

Biopsies taken from the lumbar vertebrae (L1 to L7) and femoral heads using 6 mm and 10 mm diameter cylinders, respectively, were fixed in formalin 10% (NBF—neutral buffered formalin; Thermo Scientific, Waltham, MA, USA) and stored at 4 °C. For histological preparations, the bone samples were decalcified by means of incubation in a solution of TBD-2 (Thermo Scientific, USA) with mechanical stirring for 7 (vertebrae samples) and 11 days (femoral head samples). The decalcification end point was defined as two consecutive days with negative tests for the presence of calcium in the decalcification solution supernatant. In brief, 1.0 mL of citrate–phosphate buffer (0.20 M citric acid and 0.16 M dibasic potassium phosphate, pH 3.2–3.6) and 2.5 mL of saturated ammonium oxalate were added to 0.5 mL of supernatant. After 20 min, a calcium precipitate will form in the test tube when the decalcification is still occurring. The decalcification was further confirmed by puncturing the decalcified bone biopsies with a needle to test their resistance.

The decalcified bone samples were then dehydrated in ascending alcohol concentrations before embedding the specimens in paraffin. Sections of 5 µm were cut in the anteroposterior plane on an automated microtome (HM 355S Automatic Microtome, Thermo Scientific, USA) and mounted in glass slides.

Lastly, the histological slices were deparaffinized through decreasing alcohol concentrations and stained with Hematoxylin and Eosin (H&E) (Thermo Scientific, USA) and Masson Trichrome (Bio-Optica Milano S.p.a, Milan, Italy) using standardised protocols.

### 2.5. Bone Histomorphometry

At the cortical level, the cortical porosity (Ct.Po) and cortical thickness (Ct.Th) of the lumbar vertebral and femoral head biopsies were quantified, while at the trabecular bone level, the bone volume fraction (BV/TV, %), trabecular thickness (Tb.Th, mm), trabecular separation (Tb.Sp, mm), and trabecular number (Tb.N, number/mm) of the same biopsies were quantified using the BoneJ [39] plugin of ImageJ software. For this purpose, all micrographs of the histological cuts that were stained with H&E were split into the RGB channels. A bitwise operation was performed to subtract the green channel, which was strongly staining the bone marrow area, to the red channel, roughly corresponding to the bone area and bone marrow, thus rendering an image of the bone area. The resulting representations of the bone area were thoroughly treated to remove noise and binarized for the histomorphometric evaluation.

### 2.6. Statistical Analysis

Statistical analysis was performed with the SPSS statistical software (version 23.0, SPSS, Inc., IBM Company, New York, NY, USA). The values were presented as mean ± standard deviation (SD), and there was no data exclusion. After checking for normal distributions by means of visual analysis of histograms and the Shapiro–Wilk test, data between groups in the study were analysed using ANOVA and a Student’s *t*-test. A Wilcoxon test was used for comparisons of non-parametric data. A significant difference was determined at *p* < 0.05.

## 3. Results

### 3.1. General Animal Welfare Observations

The mean body weight during the study was 53.9 ± 3.8 kg for the sham control group and 57.0 ± 4.3 kg for the experimental group. No serious complications were reported during the surgical procedure and the postoperative period. Nevertheless, from the 10th postoperative week onwards, the animals in the experimental group presented some degree of *alopecia disseminate* and behavioural changes characterised by laziness and increased sleeping times, while nevertheless preserving normophagia.

### 3.2. µCT Analysis

**Figure 1:**
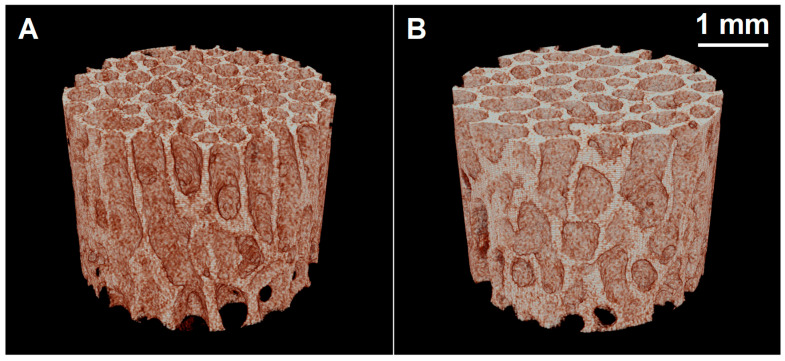
Representative micro-computed tomography 3D reconstructions of L4 vertebral bodies from (**A**) the sham control and (**B**) experimental (GC-treated and OVX sheep) groups at the 24th postoperative week.

Figure 1 presents 3D reconstructions of consecutive µCT images harvested from L4 vertebra of sheep from the sham control and experimental (GC-treated and OVX sheep) groups. Consistent decreases in the BV/TV, Tb.Th, and Tb.N and increases in the Tb.Sp and Po(tot) in the vertebral bodies or femoral head samples were measured, but without statistical significance (Table 1 and Table 2). Regarding the BMD, a consistent decrease in the experimental group was observed for L1–L7 vertebral bodies, although without statistical significance, while a statistically significant decrease was observed at the proximal femur in the experimental group (*p* ˂ 0.05).

### 3.3. Histology

**Figure 2:**
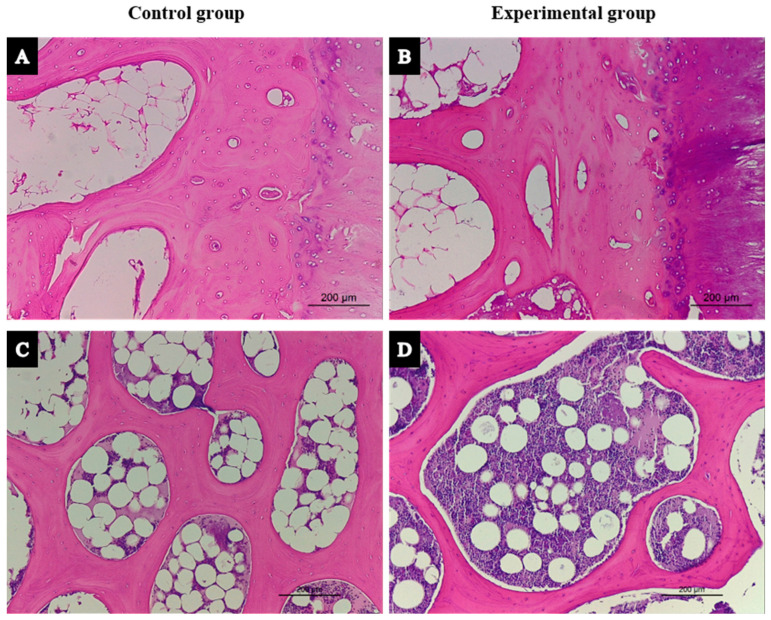
Histological differences between the two groups in the study in (**A**,**B**) the cortical and (**C**,**D**) trabecular bone tissue from vertebral biopsies (×10).

**Figure 3:**
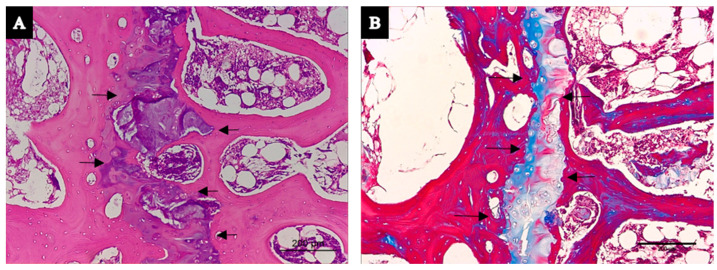
L4 vertebral biopsy with (**A**) H&E staining (**B**) and Masson Trichrome staining (×10). Identification of osteonecrosis process (arrows) in one vertebra of experimental (GC-treated and OVX sheep) group.

Morphological differences were detected at the cortical bone level of vertebral bodies, with the animals in the experimental (GC-treated and OVX sheep) group presenting with greater porosity than the healthy animals in the sham control group (Figure 2A,B). Regarding trabecular bones of the vertebral bodies, there were no evident morphological differences between the two groups (Figure 2C,D). However, in some samples of vertebral bodies of the experimental group, pathological necrosis in micro-fracture areas were observed (Figure 3A,B).

**Figure 4:**
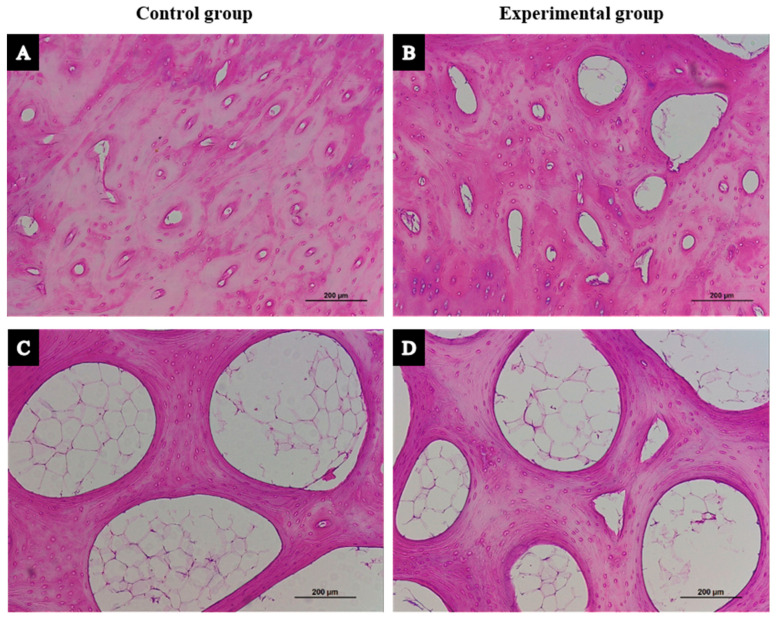
Histological differences between the sham control and experimental (GC-treated and OVX sheep) groups in (**A**,**B**) the cortical and (**C**,**D**) trabecular bone tissue of femoral heads (×10).

As in the vertebral biopsies, the bone tissue of femoral heads from the experimental group presented greater porosity than that from the sham control group (Figure 4A,B). However, no evident morphological differences were seen in trabecular bones (Figure 4C,D).

### 3.4. Bone Histomorphometry

**Figure 5:**
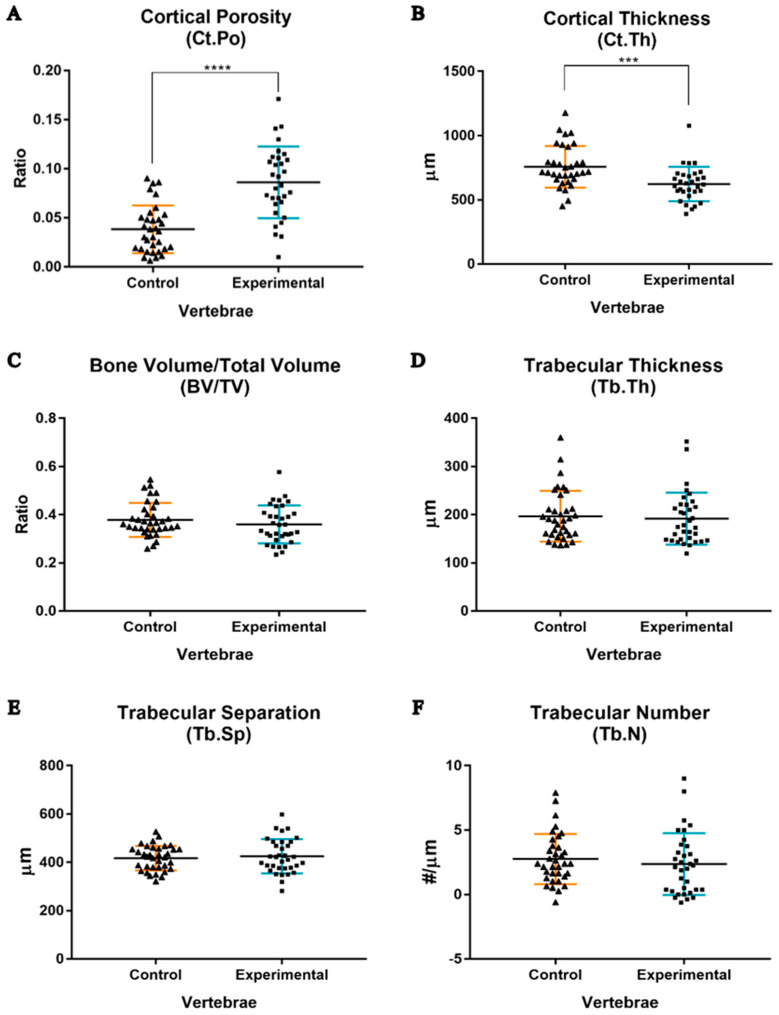
Histomorphometry analyses performed on the entirety of the cortical and trabecular bone tissue of lumbar vertebral samples. Graphical representation, with mean ± SD, of the differences in Ct.Po (**A**), Ct.Th (**B**), BV/TV (**C**), Tb.Th (**D**), Tb.Sp (**E**), and Tb.N (**F**) between the study groups. Each triangle (▲) represents a control individual sample, and each square (■) represents an experimental individual sample. Symbols denote significant differences for *p* < 0.001 (***) and *p* < 0.0001 (****).

**Figure 6:**
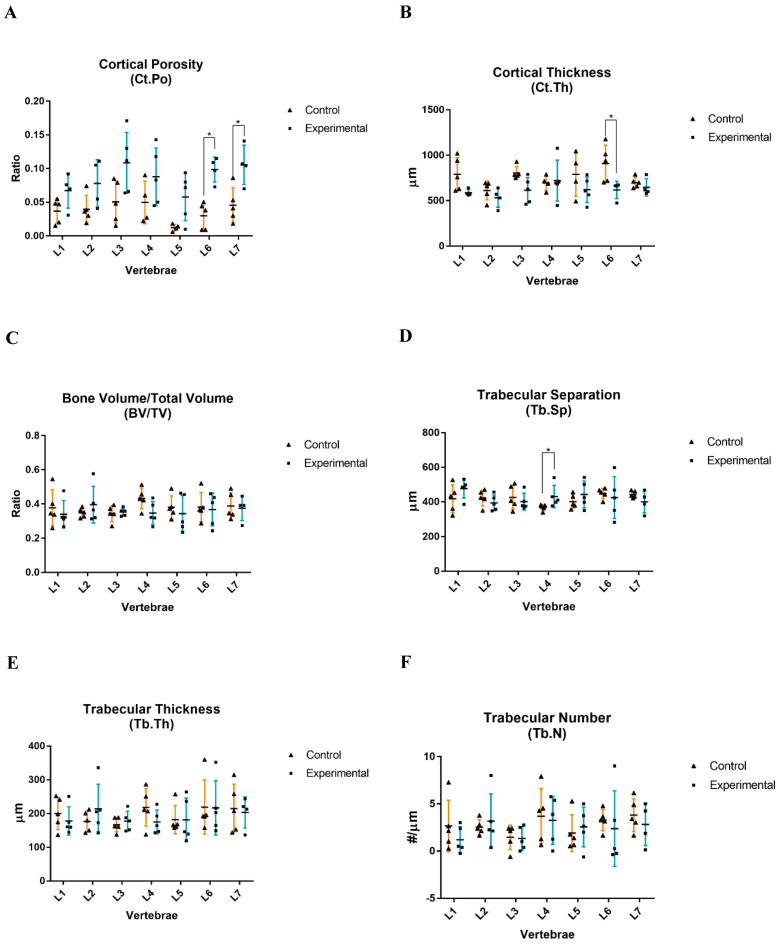
Histomorphometry analyses performed on the cortical bone of lumbar vertebral samples. Graphical representation, with mean ± SD, of the Ct.Po (**A**), Ct.Th (**B**), BV/TV (**C**), Tb.Sp (**D**), Tb.Th (**E**), and Tb.N (**F**) of the vertebrae (L1 to L7) from both study groups. Each triangle (▲) represents a control individual sample, and each square (■) represents an experimental individual sample. The (*) symbol denotes significant differences for *p* < 0.05.

Comparing the entire sham control group and experimental group at the cortical bone level, it was verified that OVX and GC administration significantly affected both the Ct.Po and Ct.Th (Figure 5A,B). The combined treatment promoted an overall Ct.Po increase from 3.8 ± 0.4% to 8.6 ± 0.7% (*p* < 0.0001) (Figure 5A) and a Ct.Th decrease from 757.6 ± 162.1 µm to 623.5 ± 134.0 µm (*p* < 0.001) in the lumbar vertebrae (Figure 5B). In particular, it was observed that the most affected vertebrae in terms of Ct.Po values were the L6 and L7, where the Ct.Po increased significantly from 3.0 ± 2.0% to 9.9 ± 1.9% and from 4.6 ± 2.6% to 10.6 ± 2.9% (*p* < 0.05), respectively (Figure 6A). Regarding the Ct.Th, significant differences were only observed in the L6 vertebra, in which there was a decrease in thickness from 908.6 ± 201.7 µm to 618.4 ± 96.0 µm (*p* < 0.05) (Figure 6B). Regarding the trabecular bone, comparing the entire sham control and experimental groups, no significant differences were observed in any of the assessed parameters (Figure 5C–F). Likewise, there were no significant differences in the evaluated parameters for each lumbar vertebra individually (Figure 6C,E,F), except for the L4 vertebra, in which the Tb.Sp increased significantly from 366.7 ± 17.6 µm to 430.8 ± 64.2 µm (*p* < 0.05) (Figure 6D).

**Figure 7:**
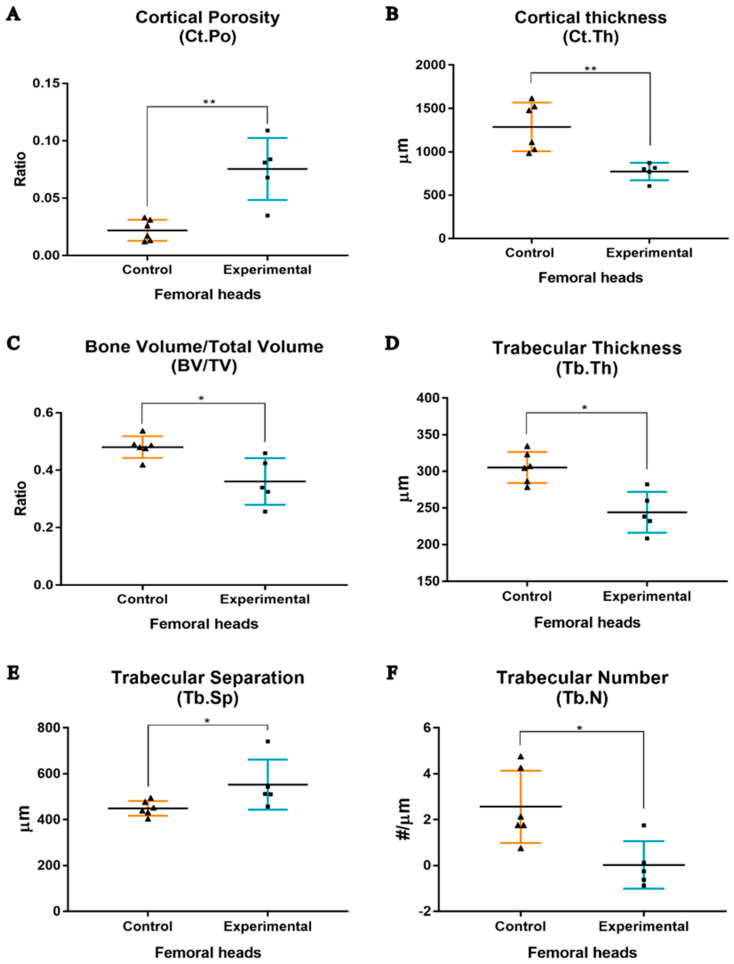
Histomorphometry analyses performed on the cortical and trabecular bone tissues of femoral head samples. Graphical representation of the differences in Ct.Po (**A**), Ct.Th (**B**), BV/TV (**C**), Tb.Th (**D**), Tb.Sp (**E**), and Tb.N (**F**) between the study groups. Each triangle (▲) represents a control individual sample, and each square (■) represents an experimental individual sample. Asterisks denote significant differences for *p* < 0.05 (*) and *p* < 0.01 (**).

In the subchondral cortical bone of the femoral heads, it was observed that the Ct.Po increased from 2.2 ± 0.4% to 7.5 ± 1.2% (*p* < 0.01) in the experimental group (Figure 7A). On the other hand, the Ct.Th decreased from 1238.7 ± 114.4 μm to 772.6 ± 45.1 μm (*p* < 0.01) (Figure 7B). In the trabecular bone analysis, the BV/TV, Tb.Th, Tb.Sp, and Tb.N were assessed, with significant differences being observed in all parameters (Figure 7C–F). Combined OVX and GC treatment promoted a reduction in bone volume from 48.1 ± 3.8% to 36.1 ± 8.1% (*p* < 0.05) (Figure 7C), a reduction in Tb.Th from 305.4 ± 21.2 μm to 244.2 ± 28.1 μm (*p* < 0.05) (Figure 7D), and a decrease in Tb.N from 2.6 ± 1.6 #/μm to 0.025 ± 1.0 #/μm (*p* < 0.05) (Figure 7F). Moreover, the Tb.Sp increased from 448.9 ± 32.6 μm to 553.0 ± 109.6 μm (*p* < 0.05) (Figure 7E).

## 4. Discussion

The present work aimed to evaluate changes in the micro-architectural parameters and composition of bone tissue at the level of the axial skeleton (focusing on lumbar vertebrae L1–L7) and appendicular skeleton (focusing on femur heads) by means of µCT and histological and bone histomorphometric analyses. In addition, individual evaluations of each of the lumbar vertebrae were carried out in order to try to determine if any of these suffered a particularly high level of bone loss, to enable later use in preclinical trials of new anti-osteoporotic drugs or spine surgical techniques in skeletons affected by osteoporosis. For that purpose, a group of sheep were bilaterally OVX and posteriorly subjected to steroid therapy for 24 weeks [37,38]. The osteoporosis induction in this animal model has exhibited similarities with the pathophysiological mechanisms that occur in both postmenopausal and GC-induced osteoporosis in humans through deficient bone formation resulting from an uncoupling of bone formation and resorption during the reversal phase [28,29,30].

In the scientific literature, several studies point out that similar OVX sheep models, associated with different exogenous GC administration protocols, have, to a greater or lesser extent, resulted in effective reductions in bone quality and bone remodelling process rates after 5.5 to 7 months [28,37,38,40,41,42] or 12 months after initiation of the bone loss induction protocol [38]. In addition, Kielbowicz et al. [43,44], focusing on the characterisation and validation of the experimental osteoporosis in GC-treated and OVX sheep, conducted a study lasting 3.7 months, while Cabrera et al. [45] performed another study with two different durations: 2 and 5 months until euthanization. It should also be noted that in some of these studies, a Ca-/vit. D-deficient diet was introduced [28,37,40,41,42,43,44], while in others, it was not [37,38,45].

Regarding the results obtained by means of µCT analysis, only the proximal femur trabecular BMD showed a statistically significant decrease after OVX and exogenous GC administration (−19.7%), and a general and consistent numerical decrease in trabecular BMD was also observed at the entire vertebral body level, although without statistical significance (−6.3%). The other µCT parameters at the vertebral and femoral levels also presented consistent changes associated with bone loss, although without statistical significance. In pre- and early perimenopausal women, slight BMD changes can generally be found. However, during the late perimenopause and early postmenopausal years, the BMD declines substantially, presenting annual rates of loss of 1.8–2.3% in the spine and 1.0–1.4% in the hip [46]. At these rates, the BMD decline could reach 7–10% in the spine and 5–7% in the hip in 5 years [46]. In a review paper, Osterhoff et al. [47] described the changes in the trabecular bone tissue during early human osteoporosis, which are characterised by reductions in bone mass and strength of this type of bone tissue, demonstrating that trabecular bone property changes are variable depending on age and anatomical site. Regarding the later changes in the cortical bone tissue with osteoporosis progression and ageing in humans, Osterhoff et al. [47] reported an increase in mineral content, porosity, and diameter of the bone cortex, associated with a degradation of mechanical properties and a decrease in cortical bone thickness.

Glucocorticoid-induced osteoporosis is the most frequent and severe form of secondary osteoporosis [48,49] and can reach 12% in the first year of exogenous GC administration if not treated [50]. Lill et al. [37,40] suggested that a BMD reduction of at least 25% is necessary for the present animal model to be characterised as an osteoporotic model. Thus, and taking the observed percentages of BMD reduction at the vertebral and proximal femur levels in the present study into account, we can consider that an osteopenia stage has been reached. Our results are also in accordance with those obtained by Schorlemmer et al. [38] and Cabrera et al. [45], who reported similar trabecular BMD reductions and trabecular micro-architecture parameter changes. In addition, more marked differences at the femur than at the spine were also obtained, in accordance with the study by Schorlemmer et al. [38], with the GC-treated and OVX sheep representing more as a model of osteopenic than osteoporotic bone. For some parameters, the results of the histomorphometric and µCT analyses are comparable, namely, for the bone volume ratio (BV/TV), separation between trabeculae (Tb.Sp), and number of trabeculae (Tb.N). The differences observed in the trabecular thickness (Tb.Th) between the two methods is, most likely, linked to the fact that when using µCT, the analysis is in 3D, while the morphometric analysis is 2D; therefore, the histological sections of the analysed trabeculae may not all be present in the same cutting plane.

Regarding the bone histomorphometric analysis, the vertebral cortical bone of the healthy sheep presented an overall average of 3.8% and 757.6 μm for the Ct.Po and Ct.Th, respectively (Figure 5). The values reported for healthy human bones are Ct.Po < 5% [51] and an average Ct.Th of around 641 μm [52]. In terms of the vertebral trabecular bone, the sheep from the sham control group presented a BV/TV of 37.8%, Tb.Th of 196.8 μm, Tb.Sp of 417.5 μm, and Tb.N of 2.575 (Figure 5), while the values reported for humans are 28.8%, 228 μm, 543 μm, and 1.46 for BV/TV, Tb.Th, Tb.Sp, and Tb.N, respectively [53]. These results from the healthy sheep in the sham control group, compared with values for humans, could be justified by the fact that the studies by Aerssens et al. [20] and McLain et al. [54] report that the forces that are generated by the large and sizable musculature supporting the spine of quadruped domestic mammals increase axial compressive stresses, thus enhancing the vertebral bone mass, which exceeds that of humans.

In the present study, a statistically significant increase in the level of Ct.Po (+226%); a statistically significant decrease in the Ct.Th (−17.7%); slight and consistent decreases, without statistical significance, in the BV/TV (−4.8%), Tb.Th (−5.0%), and Tb.N (−14.3%); and an increase in the Tb.Sp (+2.4%), also without statistical significance, were observed at the vertebral level overall following OVX and GC treatment when compared with the sham control group (Figure 5). The bone histomorphometric analysis proved that the greatest influence on the morphological characteristics in the vertebral cortical bone tissue—which constitutes the outer layer of the vertebrae body—was present at the L6 and L7 vertebrae, where statistically significant increases in porosity (+330% and +230%, respectively) and statistically significant decreases in L6′s thickness (−31.9%) were observed (Figure 6). However, the L4 vertebra was the most influenced by the treatment, in terms of the morphometric evaluation of trabecular bone tissue, based on the statistically significant increase in trabecular separation (+17.5%); L5 presented a similar pattern (+9.1%), although without statistical significance (Figure 6).

The differences between our results for sheep and human references are more disparate for the femoral heads than the vertebrae, although still comparable. Sheep presented a BV/TV of 48.1%, Tb.Th of 305.4 μm, Tb.Sp of 448.9 μm, and Tb.N of 2.6 in the sham control group (Figure 7), while the values reported in humans are 26.1%, 194 μm, 638 μm, and 1.595 for BV/TV, Tb.Th, Tb.Sp, and Tb.N, respectively [55]. These dissimilarities can possibly be explained by the difference in the type of locomotion between sheep and humans, as already mentioned. In the evaluation of bone samples obtained at the femoral head level, all measured morphometric parameters presented statistically significant changes that were induced by the treatment. Thus, in the subchondral cortical bone tissue, an increase in porosity (+341%) and decrease in thickness (−38.6%) occurred, and in trabecular bone tissue, decreases in the bone volume (−25%), thickness (−20%), and number of bone trabeculae (−96%) and an increase in trabecular separation (+23.2%), all with statistical significance, were observed. This confirms the greater sensitivity of the subchondral cortical and trabecular bone tissue at the femoral level.

The study by Müller et al. [56] in an osteoporotic sheep model showed a disturbed fibril structure at the level of the L1 vertebra in a triple-treated group—OVX, GC administration, and a special Ca- and vit. D-deficient diet—but bone loss only occurred in the form of a reduced trabecular number and thickness and cortical decline, while the quality of the residual bone was preserved. These authors refer that the preserved bone tissue properties in the osteoporotic sheep model allowed for an estimation of bone strength, which behaves similarly to the human case. In the morphometric indices, Müller et al. [56] observed more significant decreases in BT/TV and Tb.Th and increase in BS/BV than in our study. This could be explained in part by the fact that in the present study, a Ca-/vit. D/P-deficient diet was not implemented. This was because old sheep were used in the present study, since by themselves, these are referred to as a suitable model of senile osteopenia, with markedly diminished bone structure and formation and substantially augmented bone erosion [57].

Despite being useful, studies using animal models exhibit certain limitations and non-negligible biassing factors. In particular, the reduced number of animals per group and the inter-individual variability within each group, as observed herein, suggest the selection of very large cohorts for statistical validation of variables presenting minor differences or high variability. In addition, the use of sheep of different breeds and ages; the different protocols for exogenous administration of GCs with regard to their active principle, dose, route, and frequency of administration; and the durations of the studies published in the scientific literature can introduce increased variability in the obtained results and limit the possibility of comparison between studies using this animal model. It should also be noted that in the present study, there was no comparison with preoperative values within the same group, but only between the sham control and experimental (GC-treated and OVX sheep) groups at the 24th postoperative week, with the sham control group representing a physiologically healthy condition. Therefore, the possibility of a slight decrease in the morphometric parameter values within the experimental group between the pre- and postoperative period at the 24th week should not be totally excluded.

## 5. Conclusions

When looking specifically at the lumbar vertebral evaluation in studies published based on this animal model, these works usually only focus on the evaluation of one to four of the lumbar vertebrae depending on the methods used for quantifying bone tissue quality and loss. In addition, in the following phase, studies carried out in osteopenic or osteoporotic sheep, namely for vertebral augmentation in fragility compression fractures by means of percutaneous vertebroplasty or balloon kyphoplasty and implant fixation for spinal fusion, were also performed on different isolated vertebrae or groups of vertebrae, without the lumbar vertebrae having been individually assessed in terms of their response to OVX and exogenous administration of GCs.

Therefore, the originality and principal conclusion of this study are especially related to individual analyses of bone tissue’s micro-architectural and compositional characteristics in the bodies of all lumbar vertebrae—L1 to L7—in response to OVX associated with GC administration in sheep, based on µCT and histomorphometric analysis. This analysis aimed to verify whether significant differences would occur between different vertebrae, allowing us to conclude that statistically significant differences arose at the cortical bone level at L6–L7 and at the trabecular bone level at L4, demonstrating these vertebrae to be the most suitable for further preclinical and translational studies of vertebral augmentation or spinal fusion in this animal model.

## Figures and Tables

**Table 1:** Micro-architectural parameters and bone mineral density (BMD), obtained by means of micro-computed tomography at trabecular bone level of lumbar vertebrae L1 to L7 of studied groups (mean ± SD).

	Lumbar Vertebrae						
	L1	L2	L3	L4	L5	L6	L7
Sham control group							
BV/TV (%)	46.6 ± 8.1	47.9 ± 5.7	43.9 ± 5.9	44.1 ± 5.8	44.3 ± 4.7	43.6 ± 4.1	43.3 ± 8.8
BS/BV (1/mm)	17.3 ± 2.3	18.5 ± 2.5	19.3 ± 1.8	18.1 ± 1.5	16.6 ± 1.7	17.4 ± 0.8	17.8 ± 2.0
Tb.Th (mm)	0.143 ± 0.017	0.146 ± 0.018	0.137 ± 0.028	0.134 ± 0.019	0.151 ± 0.014	0.141 ± 0.020	0.139 ± 0.027
Tb.N (1/mm)	3.29 ± 0.59	3.33 ± 0.40	3.26 ± 0.86	3.39 ± 0.88	3.27 ± 0.48	3.17 ± 0.69	2.84 ± 0.67
Tb.Sp (mm)	0.36 ± 0.05	0.37 ± 0.02	0.37 ± 0.05	0.35 ± 0.05	0.38 ± 0.04	0.38 ± 0.05	0.36 ± 0.02
Po(cl) (%)	0.24 ± 0.14	0.26 ± 0.12	0.25 ± 0.21	0.20 ± 0.13	0.19 ± 0.12	0.24 ± 0.20	0.18 ± 0.13
Po(op) (%)	53.3 ± 8.2	56.2 ± 5.7	59.9 ± 6.1	52.7 ± 5.9	55.7 ± 4.8	56.3 ± 4.1	56.6 ± 9.0
Po(tot) (%)	53.4 ± 8.1	56.3 ± 5.7	58.1 ± 5.9	52.9 ± 5.8	55.7 ± 4.7	56.4 ± 4.1	56.7 ± 8.8
BMD (g/cm^3^)	0.629 ± 0.130	0.627 ± 0.106	0.640 ± 0.094	0.663 ± 0.077	0.677 ± 0.129	0.629 ± 0.080	0.640 ± 0.123
Experimental group (GC-treated and OVX sheep)							
BV/TV (%)	43.1 ± 5.5	45.6 ± 6.4	42.3 ± 4.0	42.5 ± 3.2	43.2 ± 5.5	43.9 ± 5.8	41.7 ± 4.8
BS/BV (1/mm)	18.2 ± 0.9	18.4 ± 2.8	19.6 ± 1.6	18.6 ± 0.6	17.8 ± 1.9	18.1 ± 1.7	18.4 ± 1.1
Tb.Th (mm)	0.137 ± 0.008	0.139 ± 0.018	0.132 ± 0.015	0.140 ± 0.007	0.142 ± 0.008	0.140 ± 0.011	0.143 ± 0.012
Tb.N 1/mm	3.14 ± 0.10	3.31 ± 0.64	3.25 ± 0.54	3.05 ± 0.37	3.05 ± 0.50	3.17 ± 0.63	2.93 ± 0.57
Tb.Sp (mm)	0.38 ± 0.05	0.39 ± 0.07	0.36 ± 0.03	0.40 ± 0.02	0.39 ± 0.08	0.37 ± 0.03	0.38 ± 0.05
Po(cl) (%)	0.18 ± 0.07	0.20 ± 0.09	0.21 ± 0.10	0.15 ± 0.11	0.17 ± 0.08	0.22 ± 0.18	0.15 ± 0.13
Po(op) (%)	56.8 ± 2.4	58.3 ± 6.4	57.6 ± 4.1	59.4 ± 3.3	58.2 ± 2.9	56.0 ± 5.9	58.3 ± 4.8
Po(tot) (%)	56.9 ± 2.5	58.4 ± 6.4	57.7 ± 4.0	59.5 ± 3.2	56.8 ± 5.5	56.5 ± 5.8	58.3 ± 4.8
BMD (g/cm^3^)	0.568 ± 0.013	0.607 ± 0.090	0.603 ± 0.101	0.598 ± 0.106	0.621 ± 0.116	0.626 ± 0.115	0.599 ± 0.116

**Table 2:** Micro-architectural parameters and bone mineral density (BMD), obtained by means of micro-computed tomography at trabecular bone level of femoral heads of studied groups (mean ± SD).

	Femoral Heads	
	Sham Control Group	Experimental Group (CG-Treated and OVX Sheep)
BV/TV (%)	47.2 ± 4.5	45.1 ± 3.6
BS/BV (1/mm)	32.2 ± 10.6	23.6 ± 8.0
Tb.Th (mm)	0.17 ± 0.01	0.12 ± 0.07
Tb.N 1/mm	7.02 ± 1.81	4.94 ± 2.10
Tb.Sp (mm)	0.37 ± 0.05	0.41 ± 0.05
Po(cl) (%)	0.87 ± 0.33	0.77 ± 0.45
Po(op) (%)	52.4 ± 4.5	56.6 ± 3.8
Po(tot) (%)	52.8 ± 4.5	54.9 ± 3.6
BMD (g/cm^3^)	0.785 ± 0.051	0.656 ± 0.039 *

Means after induction protocol that are followed by an asterisk differ from the mean before induction *p* ˂ 0.05 (*).

## Data Availability

The original contributions presented in this study are included in the article. Further inquiries can be directed to the corresponding author(s).
